# Supplementary material for: In-depth plasma N-glycoproteome profiling using narrow-window data-independent acquisition on the Orbitrap Astral mass spectrometer
Source: Nat Commun. 2025 Mar 13;16:2497. doi: 10.1038/s41467-025-57916-1 (PMC11906852; doi:10.1038/s41467-025-57916-1)
Supplement: Supplementary file 1 — Supplementary Information [file 41467_2025_57916_MOESM1_ESM.pdf]

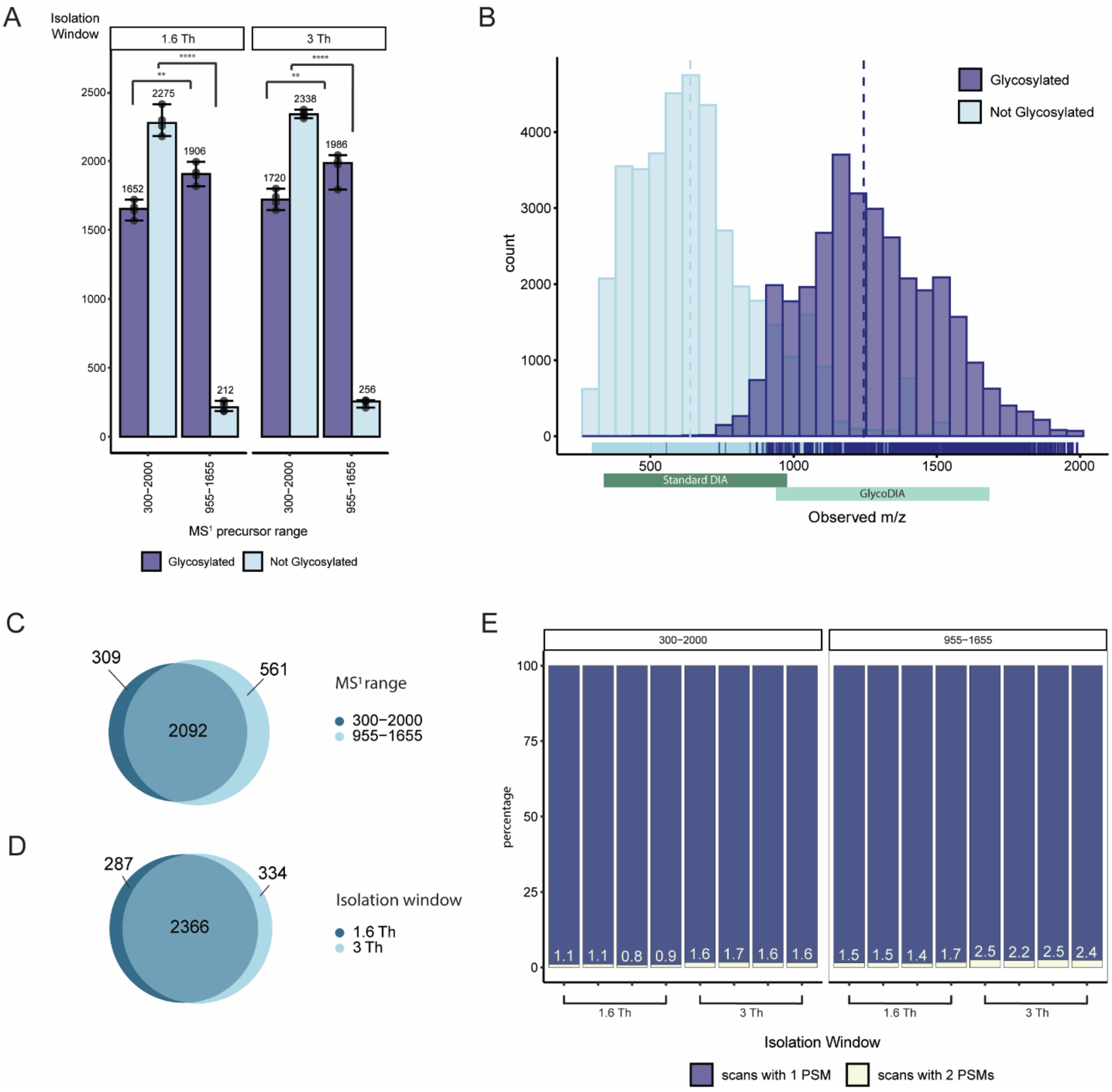

Supplemental Figure 1: Evaluation of nGlycoDIA parameters in DDA using glycopeptide-enriched plasma. A) Number of unique glycosylated PSMs (dark blue) and non-glycosylated PSMs (light blue) identified in at least two injection replicates using different MS^1 precursor ranges (x-axis) and isolation windows (left and right panels). The bar extends to the median value (n = 4, technical replicates), with the error bars indicating the minimum and maximum values, individual values are overlayed as dots. The asteriks (*) indicate statistically significant differences at p < 0.05 (*), p < 0.01 (**), p < 0.005 (***) and p < 0.001 (****) (TukeyHSD test). As can be seen, the number of non-glycosylated peptides decreased by more than 90% by changing the MS^1 range from *m/z* 300-2000 to *m/z* 955-1655, whereas the number of identified glycosylated peptides increased by 15%. **B)** Histogram comparing the precursor *m/z* values of non-glycosylated (light blue) and glycosylated (dark blue) peptides (1.6 Th isolation window and *m/z* 300-2000 MS^1 window). As can be seen, glycosylated peptides display a distincly higher *m/z* distribution compared to non-glycosylated peptides, with the here-proposed nGlycoDIA window (*m/z* 955-1655)

covering the majority of annotated glycopeptides. **C)** Venn diagram showing the overlap in cumulatively identified glycosylated peptides between a standard (*m/z* 300-2000) and narrow (*m/z* 955-1655) MS^1 range. A high overlap between the two is visible (87%), with only a small number of glycopeptides lost. **D)** Venn diagram showing the overlap in cumulatively identified glycosylated peptides between the 1.6 Th and 3 Th isolation windows (MS^1 precursor range *m/z* 955-1655), the overlap being 87%. **E)** The percentage of chimeric spectra (scans with more than 1 PSM, annotated in yellow) for each of the injection replicates (individual bars). Source data are provided as a Source Data file.

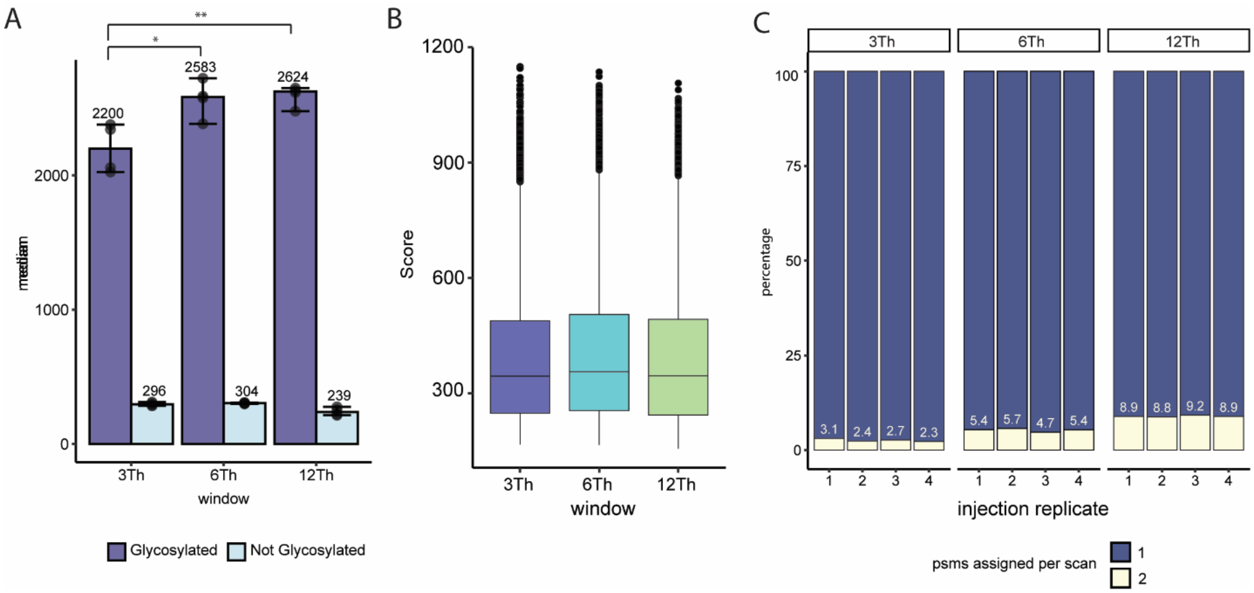

Supplemental Figure 2: Evaluation of precursor ion isolation windows in GlycoDIA. A) Number of unique glycosylated PSMs (dark blue) and non-glycosylated PSMs (light blue) identified in at least two injection replicates using different precursor isolation windows (at MS^1 range *m/z* 955-1655). The bar extends to the median value, with the error bars indicating the minimum and maximum (n = 4, technical replicates), overlayed are the indifividual data points as dots. As can be seen, the number of unique glycosylated PSMs is higher at 6 and 12 Th than at 3 Th. The asteriks (*) indicate statistically significant differences at $p < 0.05$ (*) and $p < 0.01$ (**) (TukeyHSD test). **B)** The observed Byonic Score distribution of the PSMs, showing little change with window size. The data is displayed as boxplot with center line representing the median value, upper bound at the 75th percentile and lower bound at the 25th percentile, the upper whisker extends to 1.5 * the inter-quartile range (IQR) with outliers dispicted as dots, and the lower whisker extends to the lowest value. All glycoPSMs out of the four replicates are used here, which are in total: 3Th – n = 61419, 6 Th – n = 76131, and 12 Th – n = 78045. **C)** The percentage of chimeric spectra (scans with more than 1 PSM, annotated in yellow) for each of the injection replicates (individual bars), at isolation windows of 3, 6 and 12 Th. As can be seen, the number of chimeric spectra increased with window size. Notably, isolation at 3 Th displayed a similar percentage (*i.e.* 2.6% in DIA vs 2.4% in DDA) of chimeric spectra as DDA analysis (Supplemental Figure S1E). Source data are provided as a Source Data file.

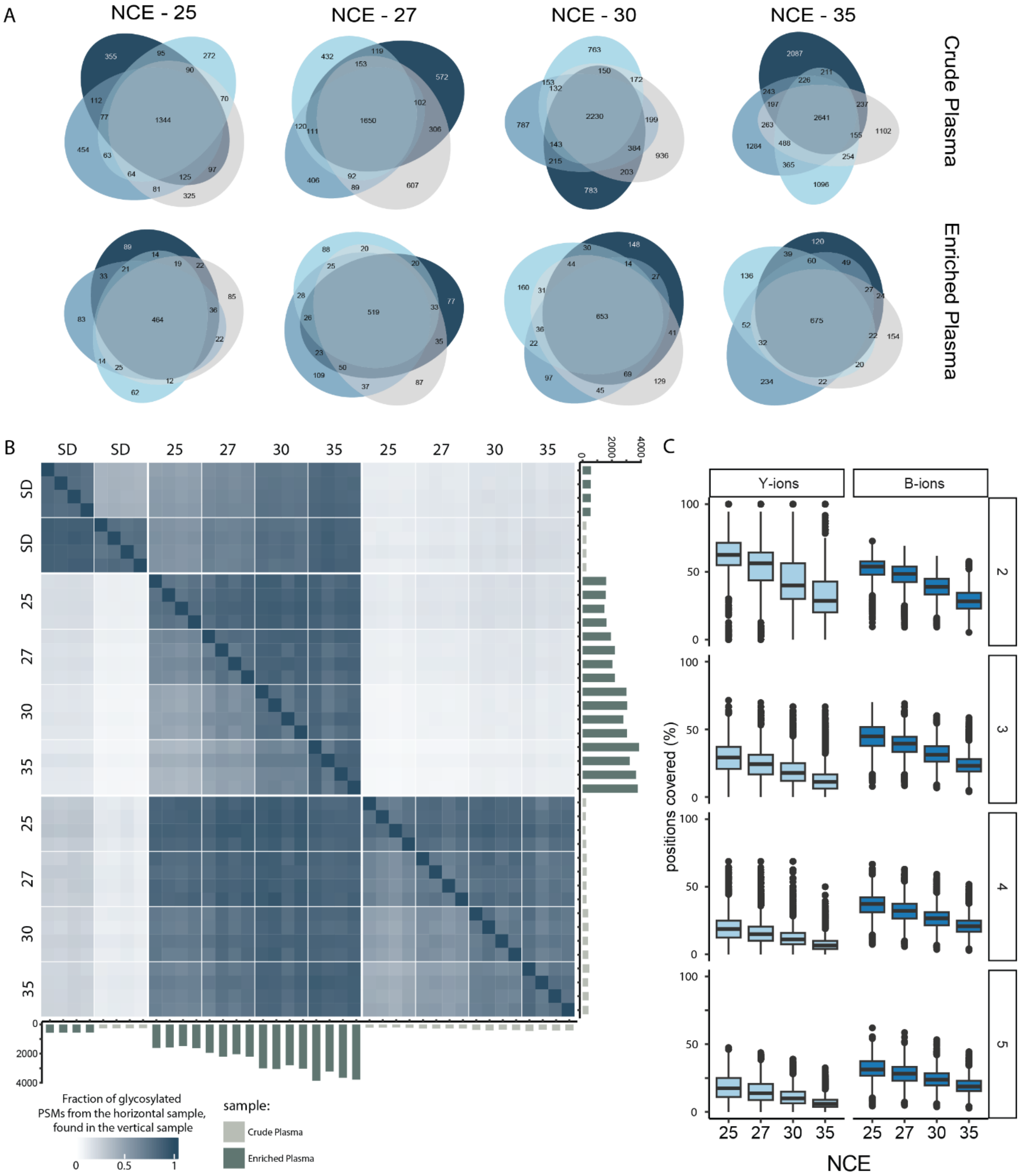

Supplemental Figure 3: Exploring the quality and reproducibility of the nGlycoDIA method. A) Venn diagrams of four replicates for each collision energy (NCE) used before applying our filtering criterium of a PSM being annotated in at least 2 out of 4 replicates in at least 1 condition. Evidently, when a PSM is annotated in at least 2 replicates, it is usually annotated in all replicates: illustrating the reproducibility of the method. **B)** Heatmap of the overlap in detected glycopeptides between samples, where the color intensity indicates the number of glycopeptides (unique glycan + modifications + peptide) from the

horizontal sample that was also identified in the vertical sample. On both axis bar plots depict the number of unique glycan PSMs that were found in each individual sample, the colors of the bars correspond to the crude or enriched samples, in light and dark green, respectively. This heatmap displays high overlap between replicates and conditions. Moreover, smaller datasets with less unique glycoPSMs are generally fully contained within the larger datasets, indicating that the increase in unique glycoPSMs is an enrichment. Values on the x- and y-axis refer to the MS/MS method used, where SD refers to the standard DIA method, and 25, 27, 30, and 35 are nGlycoDIA methods with the respective value as NCE (%). **C)** Percentage of theoretical positions covered of glycan-specific ions (Y-ions and B/oxonium-ions). The number of theoretical positions is calculated by matching the glycan composition to available structures in the GNOme database (https://pages.glycosmos.org/gnome/public/StructureBrowser.html), matching all possible fragments in all possible charge states (maximum charge = precursor charge state, mass error < 20 ppm). The 4 most abundant charge states are depicted here and separated in different panels, but demonstrate the same phenomenon, both Y- and B-ion coverage decreases as the used NCE increases. The data is displayed as boxplot with center line representing the median value, upper bound at the 75th percentile and lower bound at the 25th percentile, the upper whisker extends to the maximum value or the 1.5 * the inter-quartile range (IQR) above the 75th percentile with outliers dispicted as dots; and the lower whisker extends to the lowest value or 1.5 * the inter-quartile range (IQR) below the 25th percentile with outliers dispicted as dots. The number of PSMs used for these boxes are identical for B-ions and Y-ions with the same charge and NCE, which are the following: NCE 25 – 1936, 19634, 17880, 4680 (charge is 2, 3, 4, and 5, respectively), NCE 27 – 3062, 28071, 24389, 6288 (charge is 2, 3, 4, and 5, respectively), NCE 30 – 4960, 39804, 35373, 9319 (charge is 2, 3, 4, and 5, respectively), NCE 35 – 9663, 54085, 44295, 9418 (charge is 2, 3, 4, and 5, respectively). Source data are provided as a Source Data file.

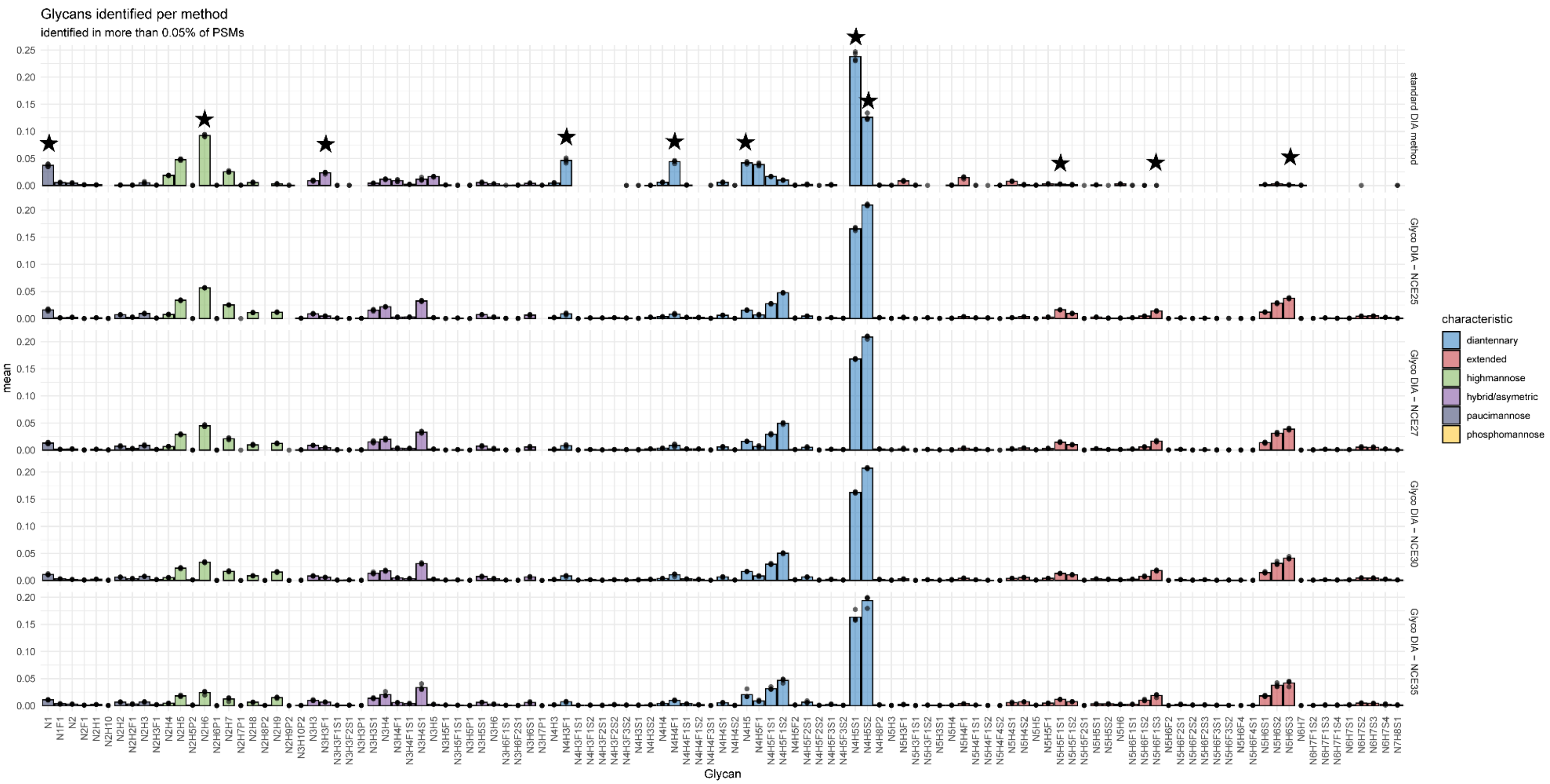

Supplemental Figure 4: Cumulative distribution of detected glycans per MS/MS method. Plotted here is the mean fraction of the total glycosylated PSMs bearing a specific glycan composition as annotated on the x-axis (n = 4, technical replicates), and the individual data points overlayed as dots. The data is filtered for a minimum abundance of 0.05% (or fraction of 0.0005). From top to bottom, the methods are standard DIA, nGlycoDIA NCE25, nGlycoDIA NCE27, nGlycoDIA NCE30, and GlycoDIA NCE35. The latter four are almost identical, while the standard DIA is quite different, with the most clearly different peaks annotated with the stars. Colors indicate glycan characteristics: diantennary complex-type (blue), extended complex-type (red), high-mannose (green), hybrid-type (purple), paucimannose (grey), and phosphomannose (yellow). Glycan compositions on the x-axis are abbreviated, where N or HexNAc is *N*-acetylhexosamine, H or Hex is hexose, F or dHex is fucose, P or Phos is phosphomannose, and S or NeuAc is *N*-acetylneuraminic acid/sialic acid. We see that there are more paucimannose glycans, and more high-mannose in the standard DIA approach, compared to the di-antennary glycans that are the most abundant in all other methods. Additionally, the

most found glycan in nGlycoDIA (N4H5S2, almost found on 20% on all PSMs) is only found on 10% of glycoPSMs in the standard DIA method. This figure clearly demonstrates that the standard DIA method created a bias in the detected glycopeptides, while collision energy itself did not introduce glycan bias. Evidently, peptides carrying paucimannose glycans are smaller than when harboring more complex glycans structure and may this better fall within the lower *m/z* window applied in the standard DIA method. Source data are provided as a Source Data file.

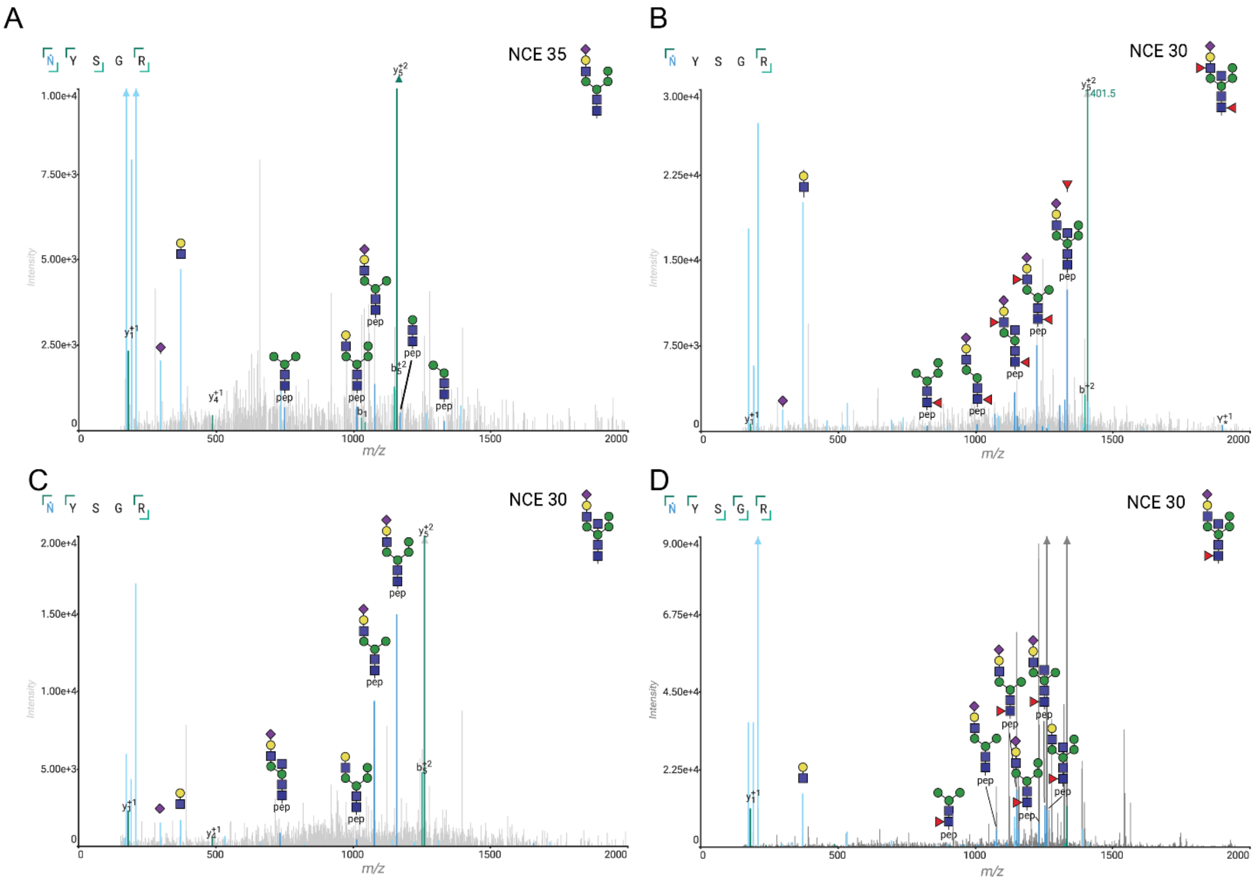

Supplemental Figure 5: annotated spectra visualizing the glycans detected on IL12B-N135. Annotated spectra of different glycans at different collision energies as depicted in the right top corner, next to the assigned glycan structure. The left top corner shows the peptide sequence with the fragment coverage of b- and y- ions. Glycan B- and Y-ions are annotated in light and dark blue, respectively. Most abundant Y- and oxonium ions are annotated with their proposed fragments. The glycan in panel **B** was originally annotated as N4H5S2, however, the Y-ions clearly show the loss of a fucose (mass loss of 146 Da) as its most abundant fragment. There are also fragments indicative of the hybrid structure and core fucosylation (the pep+N2H4F1 fragment) and we see fragments suggesting bisection, as also seen in panel **C** and **D**. This annotation, however, does not exclude other configurations or that the spectra are chimeric.

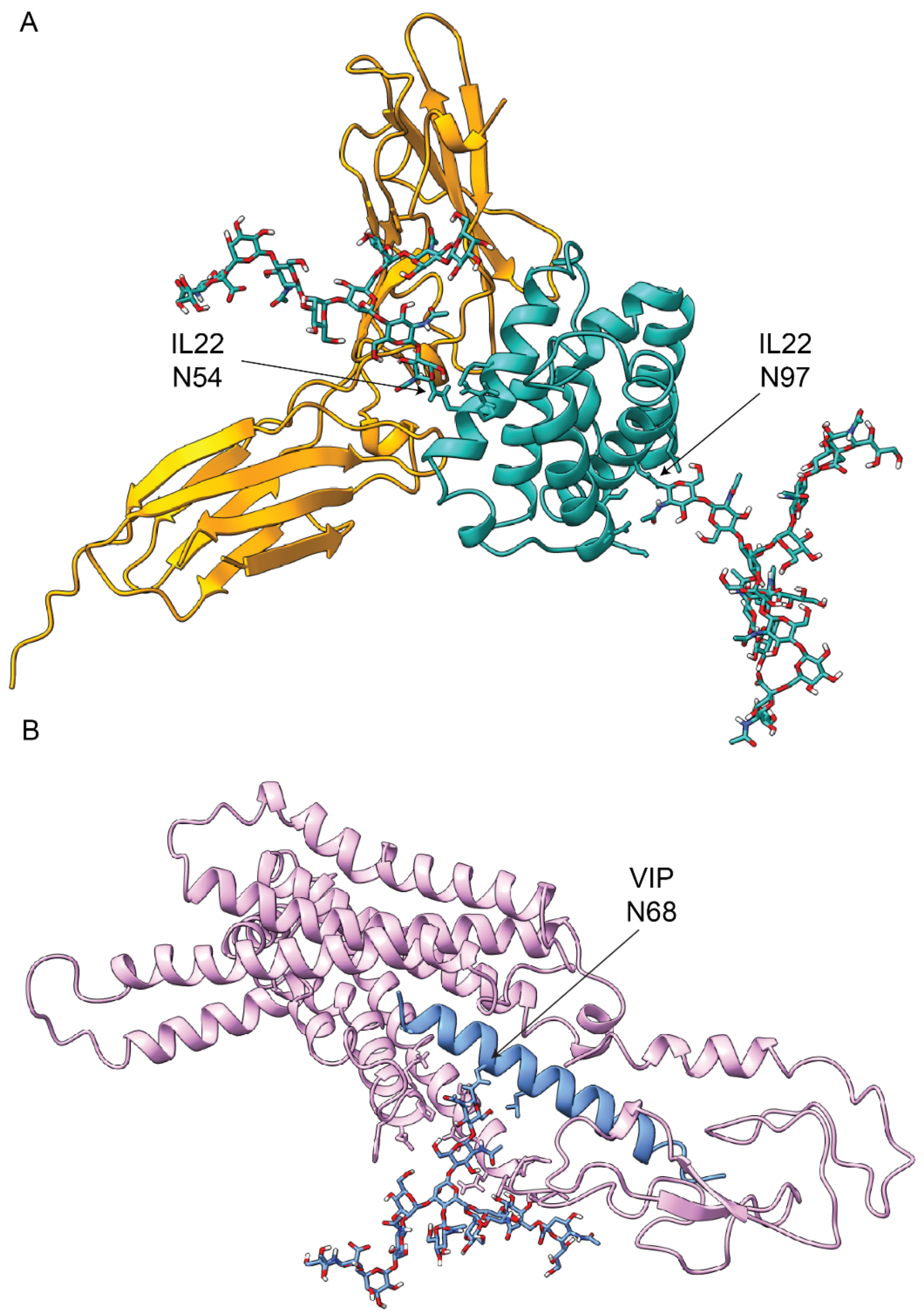

Supplemental Figure 6: Structural models of the cytokines IL22 and VIP interacting with their receptor, with modeled in the here observed glycans. A) The identified glycans are mapped on the crystal structure (PDB 3DLQ) of IL22 (turquoise) and its receptor (yellow). The glycan on N97 points away from the interaction interface, and seems not to interfere with receptor binding, while the glycan on N54 may point more towards the receptor. **B**) The identified glycan is mapped on the cryo-EM structure of VIP (blue) when bound to its receptor PAC1R (pink) (PDB 8E3Z). VIP is buried inside the extracellular domain of PAC1R, and thus this glycan may influence the binding of VIP to its receptor. Notably, the structure was resolved using non-glycosylated variants.

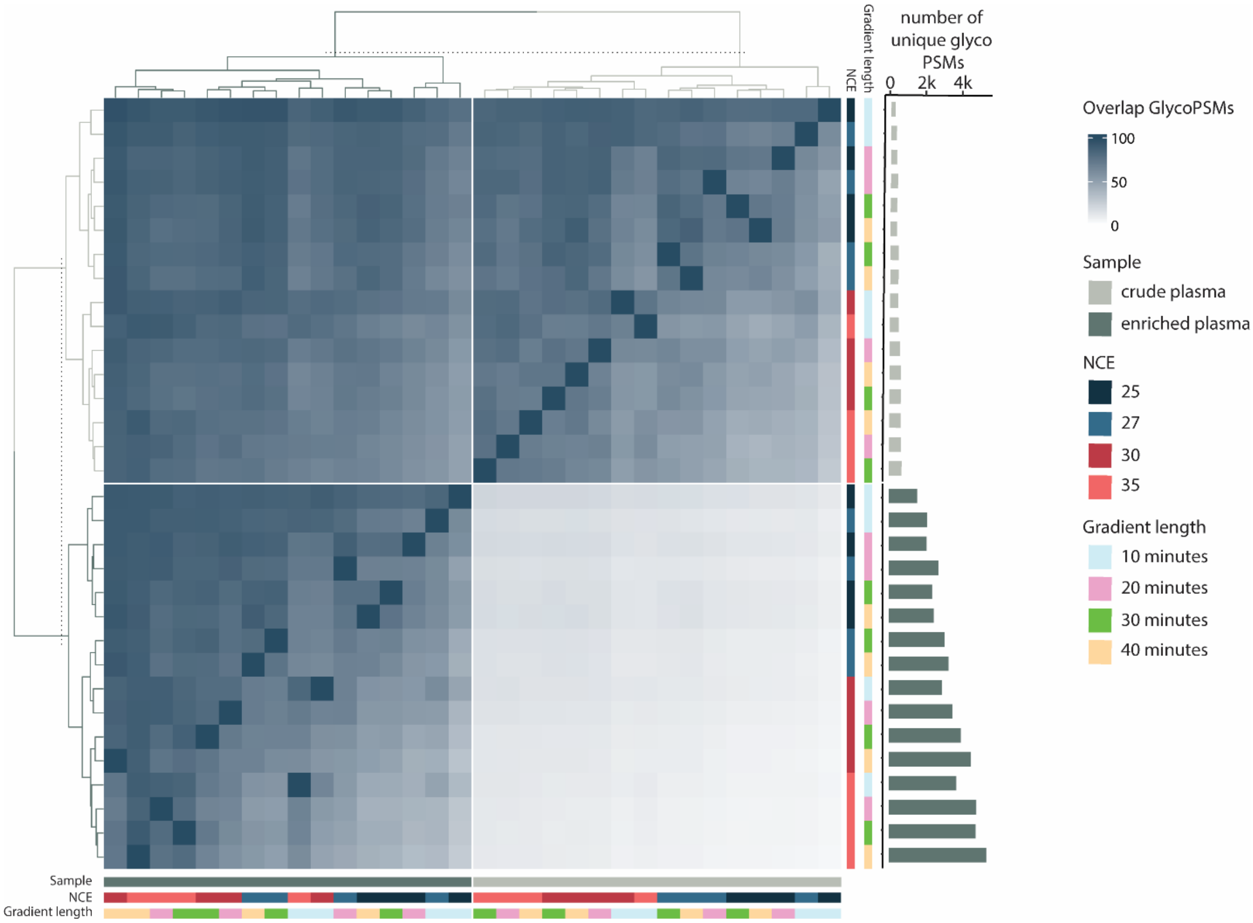

Supplemental Figure 7: **Overlap between identified glycosylated peptides using different gradient lengths and collision energies**. The heatmap color indicates the percentage of glycopeptides identified in the horizontal sample relative to the vertical sample. Sample identity is indicated by colored bars on the right and bottom sides: sample – crude and enriched plasma in light and dark green, respectively; NCE: 25, 27, 30, and 35 in dark blue, blue, dark red and salmon, respectively; and gradient length 10-, 20-, 30-, and 40-minutes in light blue, pink, green, and yellow, respectively. Samples primarily clustered based on sample identity (*e.g.* enriched or crude plasma), as well as on the total number of identified unique PSMs, as shown on the right axis. Source data are provided as a Source Data file.

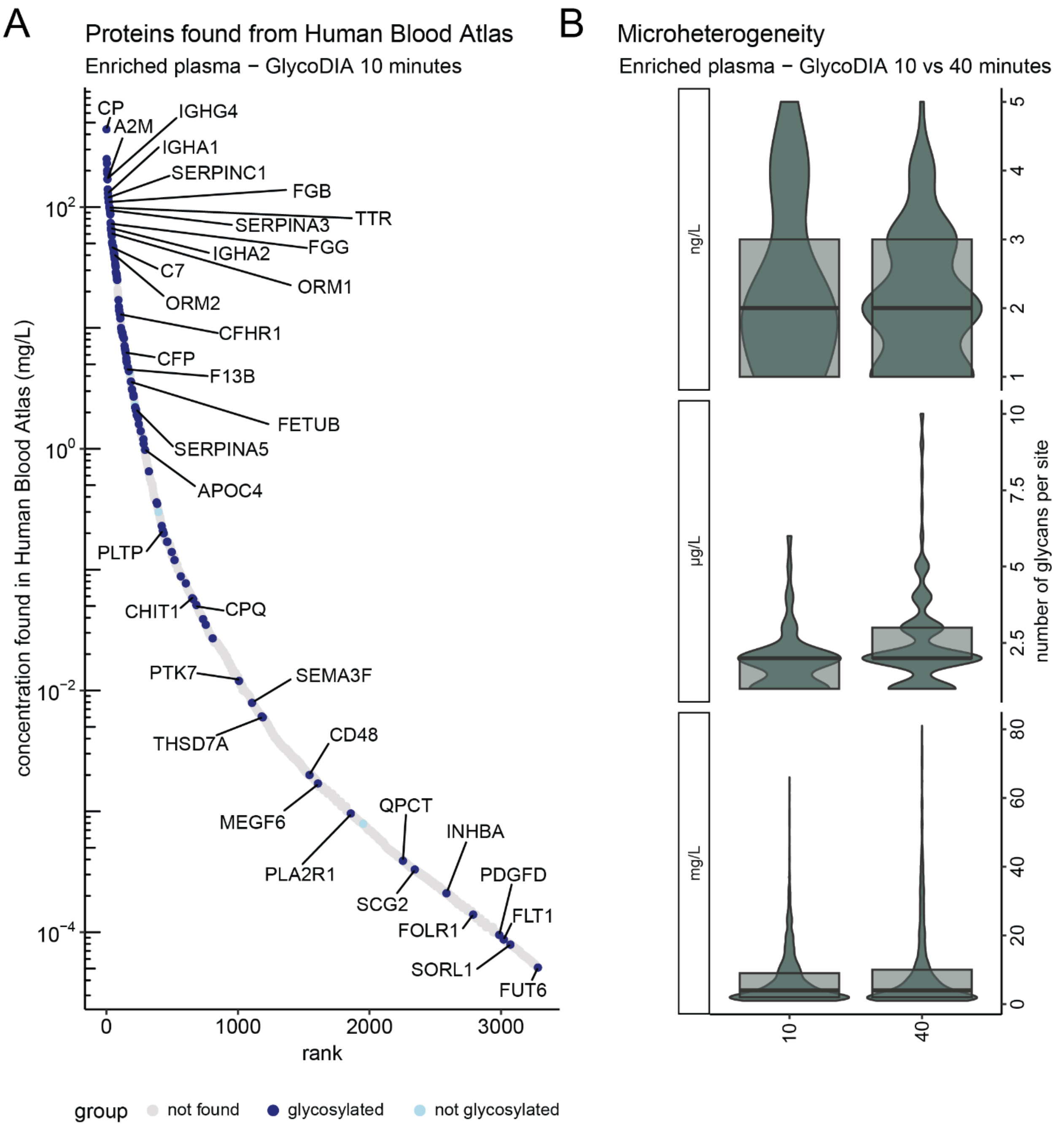

Supplemental Figure 8: Comparison of depth and microheterogeneity as observed when using a 10- or 40-minute LC-MS gradient using nGlycoDIA on a glycopeptide enriched plasma sample. **A**) The concentration of proteins identified cumulatively in the 10-minute method compared to the reported concentrations as provided in the Human Blood Atlas. The colors of the dots represent the following: grey – not identified with nGlycoDIA, light blue – non-glycosylated peptides identified with nGlycoDIA, dark blue – glycosylated peptides identified with nGlycoDIA. Even when using the 10-minute gradient still a dynamic range of 10^6 is covered. **B**) Detected glycan microheterogeneity depicted as unique glycans identified per glycosite in crude and enriched plasma, analyzed using standard nDIA and nGlycoDIA. Glycosites with only 1 psm per run were removed for this analysis. Number of glycans per site is analyzed

per individual run, and the values of the 4 injection replicates are combined for each violin. Data is grouped on the reported abundance of the proteins in the Blood Atlas, where mg/L ranges between 999 - 1 mg/L, µg/L between 999 - 1 µg/L, and ng/L below 999 ng/L. Data is displayed as a violin plot, which extends from the minimum and maximum value identified. Overlayed is a boxplot with the center line at the median value, upper bound at the 75th percentile and lower bound at the 25th percentile, without whiskers. The number of glycosites in each group was as follows: 10-minute gradient—mg/mL: n = 2508, µg/L: n = 56, ng/L: n = 21; 40-minute gradient—mg/mL: n = 3312, µg/L: n = 199, ng/L: n = 102. Note that the scales of the y-axes are different. Source data are provided as a Source Data file.

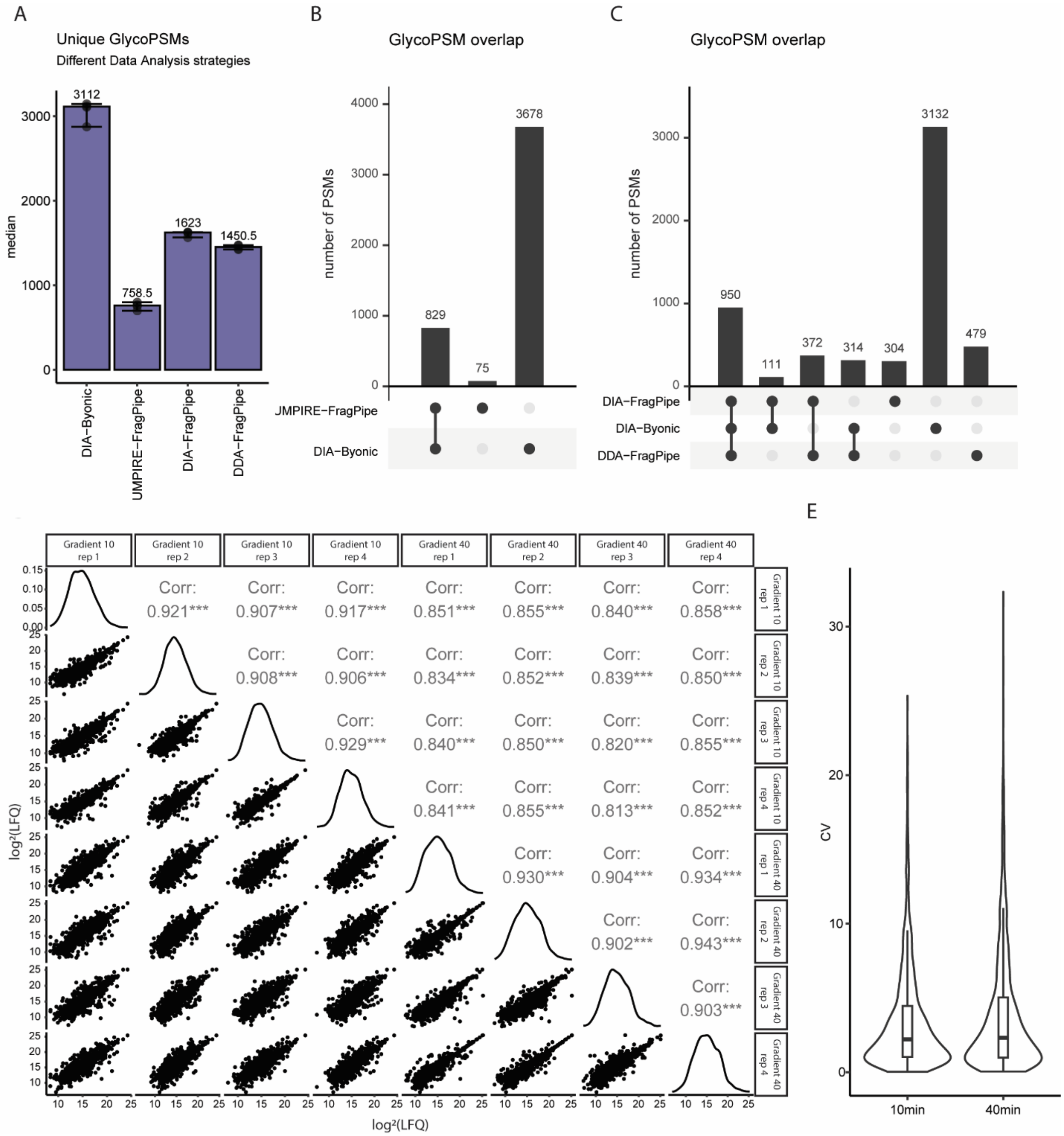

Supplemental Figure 9: Evaluation of Byonic- and FragPipe-based analysis of nGlycoDIA data. A) The number of glycosylated PSMs (glycoPSMs) in glycopeptide-enriched plasma, as reported by Byonic (DIA-Byonic), DIA-Umpire in FragPipe (Umpire-FragPipe), glyco-N-DIA in FragPipe (DIA-FragPipe), and FragPipe using a generated DDA library (DDA-FragPipe). Displayed are the medians, with the error bars indicating the minimum and maximum values (n = 4, technical replicates), individual values are overlayed as dots. **B**) Upset plots showing the overlap in glycoPSMs between Umpire-FragPipe and DIA-Byonic. **C)** Upset plots showing the overlap in glycoPSMs between DIA-FragPipe, DDA-FragPipe and DIA-Byonic. The numbers displayed in the upset plots are the total unique identified glycoPSMs across combined replicates after filtering for a minimal occurrence of 2 out of 4 replicates. The overlap between Umpire-FragPipe and DIA-

Byonic is 92%, while the overlap between DIA-FragPipe and DIA-Byonic is 61%. **D)** Scatterplots of the log2-transformed normalized precursor quantities (LFQ) as quantified by DIA-NN within FragPipe in four replicates of nGlycoDIA runs on glycopeptide enriched plasma using 10- and 40-minute gradients (bottom panels). The correlation and Pearson Coefficients are high (upper panels), both between replicates ($R > 0.9$) and between the different gradient lengths ($R > 0.8$). The diagonal shows the density plot of the log2-transformed precursor quantities. **E)** Violin plot showing the coefficient of variation (CV) in the 10- and 40-minute method. CV's are calculated from four injection replicates, with a median CV of 2.2% and 2.3% for the 10-, and 40-minute method, respectively. This indicates highly robust quantification. The violin plots extend from the minimum to maximum values, the overlayed boxplot have the median as the center line, upper bound at the 75th percentile and lower bound at the 25th percentile, the upper whisker extends to 1.5* inter-quartile range above the 75th percentile, the lower whisker extends to the minimum value. The CV's of 1197 and 1385 quantified glycopeptides are used for the 10- and 40-minute method, respectively. Source data are provided as a Source Data file.
