# Supplementary material for: In-depth plasma N-glycoproteome profiling using narrow-window data-independent acquisition on the Orbitrap Astral mass spectrometer
Source: Nat Commun. 2025 Mar 13;16:2497. doi: 10.1038/s41467-025-57916-1 (PMC11906852; doi:10.1038/s41467-025-57916-1)
Supplement: Supplementary file 2 — Reporting Summary [file 41467_2025_57916_MOESM2_ESM.pdf]

nature portfolio

Corresponding author(s): A.J.R. Heck (a.j.r.heck@uu.nl)

Last updated by author(s): 17/02/2025

Reporting Summary

Nature Portfolio wishes to improve the reproducibility of the work that we publish. This form provides structure for consistency and transparency in reporting. For further information on Nature Portfolio policies, see our Editorial Policies and the Editorial Policy Checklist.

Statistics

For all statistical analyses, confirm that the following items are present in the figure legend, table legend, main text, or Methods section.

n/a	Confirmed	
☐	☒	The exact sample size (*n*) for each experimental group/condition, given as a discrete number and unit of measurement
☐	☒	A statement on whether measurements were taken from distinct samples or whether the same sample was measured repeatedly
☐	☒	The statistical test(s) used AND whether they are one- or two-sided<br>*Only common tests should be described solely by name; describe more complex techniques in the Methods section.*
☒	☐	A description of all covariates tested
☒	☐	A description of any assumptions or corrections, such as tests of normality and adjustment for multiple comparisons
☐	☒	A full description of the statistical parameters including central tendency (e.g. means) or other basic estimates (e.g. regression coefficient) AND variation (e.g. standard deviation) or associated estimates of uncertainty (e.g. confidence intervals)
☐	☒	For null hypothesis testing, the test statistic (e.g. *F*, *t*, *r*) with confidence intervals, effect sizes, degrees of freedom and *P* value noted<br>*Give P values as exact values whenever suitable.*
☒	☐	For Bayesian analysis, information on the choice of priors and Markov chain Monte Carlo settings
☒	☐	For hierarchical and complex designs, identification of the appropriate level for tests and full reporting of outcomes
☐	☒	Estimates of effect sizes (e.g. Cohen's *d*, Pearson's *r*), indicating how they were calculated

Our web collection on statistics for biologists contains articles on many of the points above.

Software and code

Policy information about availability of computer code

Data collection	PMI-Byonic (v5.5.2, protein metrics).
Data analysis	Analysis scripts were written in R (v4.3.1) (Supplementary Data File 12). The package ComplexHeatmap (v 2.20.0) was used for heatmaps and clustering, with confidence intervals being calculated using the rcompanion (v 2.4.36) package, other plots were made using ggplot2 (v 3.5.1), ggpubr (v 0.6.0), ggrepel (v 0.9.5), GGally (v 2.2.1), UpSetR (v 1.4.0), eulerr (v 7.0.2). Other used packages are: tidyverse (v 2.0.0), seqinr (v 4.2-36), openxlsx (v 4.2.5.2), scales (v 1.3.0). Additionally Annotator (v 0.2.2 https://github.com/snijderlab/annotator) was used for annotation of spectra, and an in-house written rust script was used to access the annotator algorithm on all PSMs (Supplementary Data File 13), using the rustyms package (https://github.com/snijderlab/rustyms).

For manuscripts utilizing custom algorithms or software that are central to the research but not yet described in published literature, software must be made available to editors and reviewers. We strongly encourage code deposition in a community repository (e.g. GitHub). See the Nature Portfolio guidelines for submitting code & software for further information.

Data

Policy information about availability of data

All manuscripts must include a data availability statement. This statement should provide the following information, where applicable:

- Accession codes, unique identifiers, or web links for publicly available datasets
- A description of any restrictions on data availability
- For clinical datasets or third party data, please ensure that the statement adheres to our policy

All raw data files and Byonic output files used in this study has been reposited to the MASSive and ProteomeXchange repository and made publicly available with the following identifier: MSV000095471 and PXD054333, for MASSive and ProteomeXchange, respectively.

Research involving human participants, their data, or biological material

Policy information about studies with human participants or human data. See also policy information about sex, gender (identity/presentation), and sexual orientation and race, ethnicity and racism.

Reporting on sex and gender	Sample used here is a commercial pooled human plasma sample (VisuCon-F Normal Donor Set (EFNCP0125), Affinity Biologicals) of 20 donors. Information on sex, gender, ethnicity or other factors is not specified. The purpose of this paper is method development and there are no clinical findings presented here.
Reporting on race, ethnicity, or other socially relevant groupings	Sample used here is a commercial pooled human plasma sample (VisuCon-F Normal Donor Set (EFNCP0125), Affinity Biologicals) of 20 donors. Information on sex, gender, ethnicity or other factors is not specified. The purpose of this paper is method development and there are no clinical findings presented here.
Population characteristics	Sample used here is a commercial pooled human plasma sample (VisuCon-F Normal Donor Set (EFNCP0125), Affinity Biologicals) of 20 donors. Information on sex, gender, ethnicity or other factors is not specified. The purpose of this paper is method development and there are no clinical findings presented here.
Recruitment	NA
Ethics oversight	NA

Note that full information on the approval of the study protocol must also be provided in the manuscript.

Field-specific reporting

Please select the one below that is the best fit for your research. If you are not sure, read the appropriate sections before making your selection.

☒ Life sciences ☐ Behavioural & social sciences ☐ Ecological, evolutionary & environmental sciences

For a reference copy of the document with all sections, see nature.com/documents/nr-reporting-summary-flat.pdf

Life sciences study design

All studies must disclose on these points even when the disclosure is negative.

Sample size	For this study we chose to do each method with four replicates, to access reproducibility of the method and to allow for statistical interpretation (spread, means, ect.).
Data exclusions	some LC-MSMS runs were excluded when partial sample injection occured, which was caused by our LC-MSMS sample handling: 12 uL of sample was added to a glass vial and served for 10 injections of 1 uL. Due to sample evaporation the 10th injection from a single vial did not always inject the full sample, which could be identified by the lack of signal in these runs. This occurred in 2 samples (Enriched Plasma - NCE 35 - 30 minute - 03; and Enriched Plasma - NCE 35 - 40 minute - 02) ,and these conditions were rerun to complete the four replicates.
Replication	4 injection replicates per condition (method), each replicate was used in the data-analysis and is contained in the median, means, and CI's described in the manuscript. All attempts of replication were successful, except in the situation described above when partial sample injection occurred due to sample evaporation.
Randomization	Methods (both LC gradient duration and MS method) were randomized to decrease dilution effect of repetitive injection from a single vial.
Blinding	Blinding was not applicable because all injection derive from the same plasma sample.

Reporting for specific materials, systems and methods

We require information from authors about some types of materials, experimental systems and methods used in many studies. Here, indicate whether each material, system or method listed is relevant to your study. If you are not sure if a list item applies to your research, read the appropriate section before selecting a response.

Materials & experimental systems

n/a	Involved in the study
☒	☐ Antibodies
☒	☐ Eukaryotic cell lines
☒	☐ Palaeontology and archaeology
☒	☐ Animals and other organisms
☒	☐ Clinical data
☒	☐ Dual use research of concern
☒	☐ Plants

Methods

n/a	Involved in the study
☒	☐ ChIP-seq
☒	☐ Flow cytometry
☒	☐ MRI-based neuroimaging

Plants

Seed stocks

Report on the source of all seed stocks or other plant material used. If applicable, state the seed stock centre and catalogue number. If plant specimens were collected from the field, describe the collection location, date and sampling procedures.

Novel plant genotypes

Describe the methods by which all novel plant genotypes were produced. This includes those generated by transgenic approaches, gene editing, chemical/radiation-based mutagenesis and hybridization. For transgenic lines, describe the transformation method, the number of independent lines analyzed and the generation upon which experiments were performed. For gene-edited lines, describe the editor used, the endogenous sequence targeted for editing, the targeting guide RNA sequence (if applicable) and how the editor was applied.

Authentication

Describe any authentication procedures for each seed stock used or novel genotype generated. Describe any experiments used to assess the effect of a mutation and, where applicable, how potential secondary effects (e.g. second site T-DNA insertions, mosiacism, off-target gene editing) were examined.
