# Supplementary material for: In-depth plasma N-glycoproteome profiling using narrow-window data-independent acquisition on the Orbitrap Astral mass spectrometer
Source: Nat Commun. 2025 Mar 13;16:2497. doi: 10.1038/s41467-025-57916-1 (PMC11906852; doi:10.1038/s41467-025-57916-1)
Supplement: Supplementary file 3 — Description of Additional Supplementary Files [file 41467_2025_57916_MOESM3_ESM.pdf]

Description of Additional Supplementary Files

File name: Supplementary Data 1

Description: Glycan database used in the Byonic searches.

File name: Supplementary Data 2

Description: Byonic score cut-off values for each run, used in 1% peptide FDR filtering.

File name: Supplementary Data 3

Description: Zip-folder containing the R-script (‘SupplementalFile_S12.R’) used for all data analysis, plots and statistical tests, and a folder (‘UsedFiles’) which contains the downloaded data from the protein atlas (‘proteinatlas.tsv’) and GNO identifiers for glycan compositions (‘GNOtomatch.csv’).

File name: Supplementary Software 1

Description: Zip-folder containing the ‘multi-annotator’ tool used for ion-coverage calculations (used for supplementary figure S3C). The folder includes a readme-file, specifying how to install and run the multi-annotator and a folder with demo data.
